# Determination of Patient Sentiment and Emotion in Ophthalmology: Infoveillance Tutorial on Web-Based Health Forum Discussions

**DOI:** 10.2196/20803

**Published:** 2021-05-17

**Authors:** Anne Xuan-Lan Nguyen, Xuan-Vi Trinh, Sophia Y Wang, Albert Y Wu

**Affiliations:** 1 Faculty of Medicine McGill University Montreal, QC Canada; 2 Department of Computer Science McGill University Montreal, QC Canada; 3 Department of Ophthalmology Byers Eye Institute Stanford University Palo Alto, CA United States

**Keywords:** sentiment analysis, emotions analysis, natural language processing, online forums, social media, patient attitudes, medicine, infodemiology, infoveillance, digital health

## Abstract

**Background:**

Clinical data in social media are an underused source of information with great potential to allow for a deeper understanding of patient values, attitudes, and preferences.

**Objective:**

This tutorial aims to describe a novel, robust, and modular method for the sentiment analysis and emotion detection of free text from web-based forums and the factors to consider during its application.

**Methods:**

We mined the discussion and user information of all posts containing search terms related to a medical subspecialty (oculoplastics) from MedHelp, the largest web-based platform for patient health forums. We used data cleaning and processing tools to define the relevant subset of results and prepare them for sentiment analysis. We executed sentiment and emotion analyses by using IBM Watson Natural Language Understanding to generate sentiment and emotion scores for the posts and their associated keywords. The keywords were aggregated using natural language processing tools.

**Results:**

Overall, 39 oculoplastic-related search terms resulted in 46,381 eligible posts within 14,329 threads. Posts were written by 18,319 users (117 doctors; 18,202 patients) and included 201,611 associated keywords. Keywords that occurred ≥500 times in the corpus were used to identify the most prominent topics, including specific symptoms, medication, and complications. The sentiment and emotion scores of these keywords and eligible posts were analyzed to provide concrete examples of the potential of this methodology to allow for a better understanding of patients’ attitudes. The overall sentiment score reflects a positive, neutral, or negative sentiment, whereas the emotion scores (anger, disgust, fear, joy, and sadness) represent the likelihood of the presence of the emotion. In keyword grouping analyses, medical signs, symptoms, and diseases had the lowest overall sentiment scores (−0.598). Complications were highly associated with sadness (0.485). Forum posts mentioning body parts were related to sadness (0.416) and fear (0.321). Administration was the category with the highest anger score (0.146). The top 6 forum subgroups had an overall negative sentiment score; the most negative one was the *Neurology* forum, with a score of −0.438. The *Undiagnosed Symptoms* forum had the highest sadness score (0.448). The least likely fearful posts were those from the *Eye Care* forum, with a score of 0.260. The overall sentiment score was much more negative before the doctor replied. The anger, disgust, fear, and sadness emotion scores decreased in likelihood, whereas joy was slightly more likely to be expressed after doctors replied.

**Conclusions:**

This report allows physicians and researchers to efficiently mine and perform sentiment analysis on social media to better understand patients’ perspectives and promote patient-centric care. Important factors to be considered during its application include evaluating the scope of the search; selecting search terms and understanding their linguistic usages; and establishing selection, filtering, and processing criteria for posts and keywords tailored to the desired results.

## Introduction

Understanding patient attitudes and expectations toward health care is an important component of promoting patient-centric care and patient satisfaction. However, studies have shown that physicians have difficulties in understanding patients’ health beliefs and concerns [1]. Strategies to improve the understanding of patient attitudes have traditionally required the development of specialized survey instruments, which may nonetheless be limited in scope, or focus groups, which can be very time consuming and laborious [2].

The internet has now become a rich additional source of information regarding patients’ attitudes and expectations toward health care. Recent decades have seen a rapid increase in internet engagement, with an estimated 5 billion people using mobile devices [3], and more than half of the global population actively using the internet [4]. In 2012, 72% of American internet users sought health information on the web [5] and many also increasingly expressed their medical concerns on the web [6,7]. These web-based communication outlets include social networks (eg, Facebook, Twitter, or Instagram), doctor review websites (eg, Healthgrades, Vitals, or RateMDs), and health web forums (eg, MedHelp, Health245, or Patient info). Analyzing people’s health-related queries and reports on the internet to better inform public health and public policy is an increasingly popular field known as infoveillance [8]. Although Twitter is a common and popular platform based on which many infoveillance studies are conducted, its space-limited format contrasts with web-based health forums, which are a particularly rich resource for understanding patient attitudes toward medical issues by supporting patients in directly seeking medical advice, sharing their medical experiences, and discussing their symptoms at length [9-15].

Understanding unstructured clinical data on social media requires natural language processing (NLP), a well-established branch of artificial intelligence that has been applied in a variety of fields and has emerging applications in medicine [16,17]. Sentiment and emotion analyses, which are subbranches of NLP, can identify and quantify positive, neutral, and negative sentiments and can detect emotions such as anger, disgust, fear, joy, and sadness in free text [18,19]. The data mining and sentiment analysis of social media, especially web-based medical discussion forums, can provide a fast and effective way to better understand patients’ attitudes, expectations, and experiences [18], which can better guide patient-centric care [20]. The literature shows that health care professionals can, with the sentiment analysis of web-based medical forums, discover new outlooks of patient issues and recurrent complications related to specific treatment uses and drugs [19,21,22] and administrative burden and access to care [23]. By analyzing forum posts, physicians can further understand patients’ attitudes and experiences and assess their needs and concerns, which can result in better patient-centric care [24].

We examined all oculoplastics-related posts on MedHelp, which included questions from patients and replies written by patients and doctors. Oculoplastics is a subspecialty in ophthalmology that involves the eyelids, face, tear ducts, and orbit and is both highly specialized and interdisciplinary as a clinical domain, often at the intersection of ophthalmology, plastic surgery, dermatology, and otolaryngology. Our study illustrates the challenges of identifying and distinguishing text related to specialized medical subdomains, such as ophthalmology, in the context of patient-centric idiomatic language and of web-based discussion forum analysis, where the relevance of text must be filtered on multiple structural levels and physician and patient posts must be distinguished from physicians’ posts. We provide all scripts and describe a detailed approach toward web-based patient forum sentiment analysis, which includes data collection; rigorous data processing, cleaning, and selection; and in-depth data analysis. This methodology allows for a variety of applications, notably the identification and analysis of the main topics related to the chosen field (eg, symptoms, complications, and medication) and their associated quantified sentiment (positive, neutral, or negative) and the likelihood of the presence of certain emotions (joy, anger, disgust, sadness, and fear). This methodology can also be used as a means to measure patient satisfaction and perspective by comparing patients’ sentiment and emotions before, during, and after their interaction with health care professionals. This paper aims to guide physicians and researchers to mine and perform sentiment analysis on web-based clinical data in a chosen field and highlights the challenges and approaches to consider in the process.

## Methods

### Data Source and Study Population

Founded in 1994, MedHelp is the world’s largest web-based health community [25]. With more than 15 million visits per month, it allows users (patients and doctors) to discuss issues related to various health and wellness topics on a daily basis [18]. Currently, this platform contains 299 official support communities, including a wide variety of well-established medical discussion forums. The main oculoplastic discussion forum is the *Eye Care Community*, which encourages patients to discuss eye-related issues. Another vision-related forum was the *Ask a Doctor*-*Eye Care Forum*, which benefited from a collaboration with ophthalmologists from the American Academy of Ophthalmology from 2007 to 2014 [25,26]. In addition to these forums, MedHelp has more than 1000 user-made groups.

Each community or group, also referred to as *a forum*, encompasses various discussion *threads*. Discussion threads comprise a question asked by a user (the initial *post*), followed by replies written by individual users, which are also considered *posts* [19].

### Approach to Data Extraction

**Figure 1:**
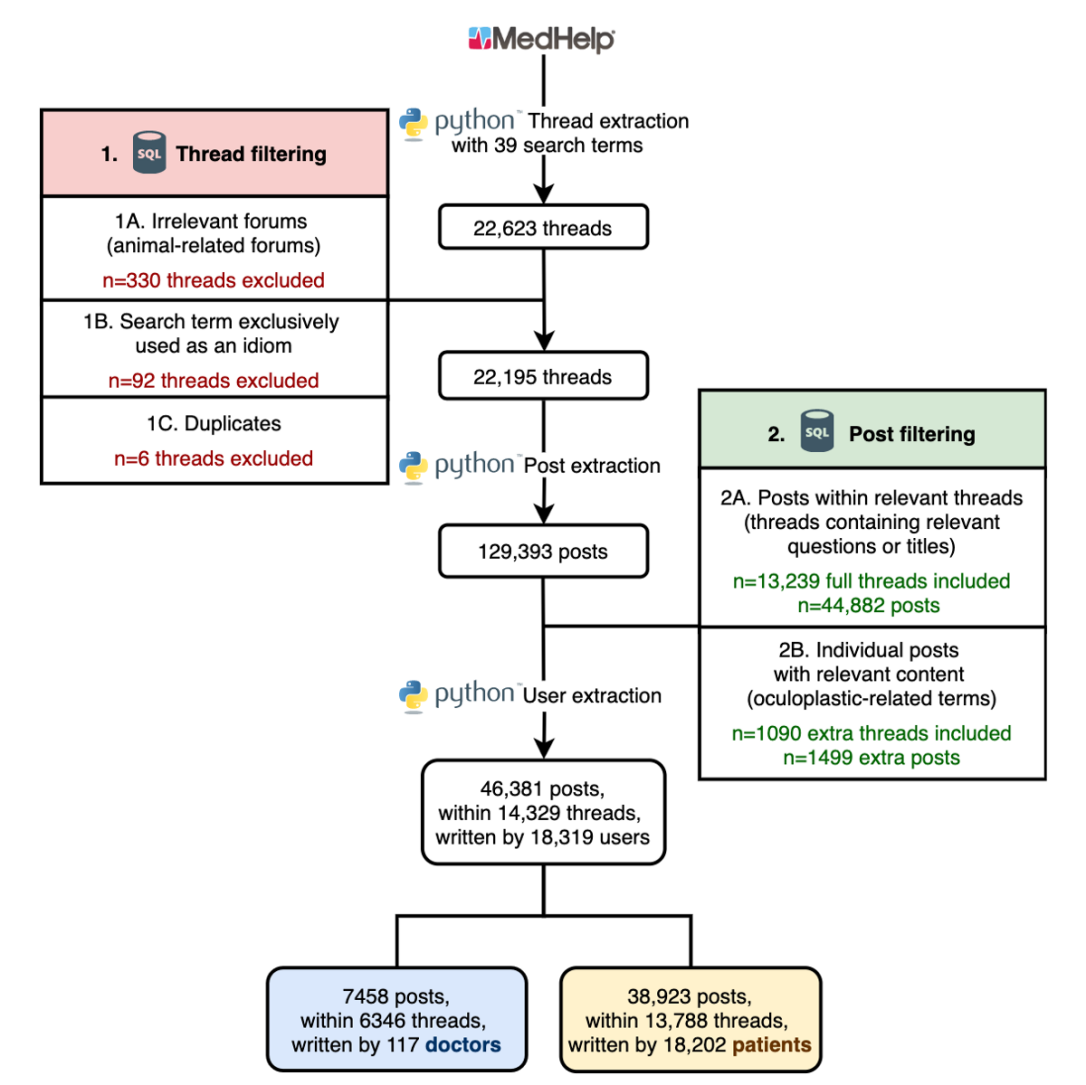
Flowchart for the data extraction of discussion threads and posts on web-based medical forums. SQL: Structured Query Language.

The approach to data extraction from MedHelp is summarized in Figure 1. Discussion threads related to oculoplastic surgery were identified from MedHelp using a list of oculoplastics-relevant search terms created by consensus between 2 specialized ophthalmologists, AYW and SYW, and AXN (Multimedia Appendix 1).

Each discussion thread was parsed using a Python script (Python Software Foundation, version 3.8.6) [27] and the Python package *Beautiful Soup* [28] to yield the full text of each post (including the initial question and all replies) and the relevant metadata, including the MedHelp user for each post and the forum that each thread belonged to.

An initial review of the search results demonstrated that not all results appeared to be relevant, and it was noted that the details of the exact algorithm used by MedHelp’s proprietary search engine could not be known. Thus, we performed additional filtering of the search results to remove irrelevant discussion threads. Threads in animal forums, duplicate threads, and threads where the search terms were mentioned in purely idiomatic ways were removed.

In addition, we noted that many threads were returned as search results because search terms appeared in different posts within the same thread, for example, the search term “double eye lid” could return a thread containing the use of “double,” “eye,” and “lid” in separate posts, which could result in many irrelevant posts.

Therefore, to further filter the posts to include those that were most highly relevant to oculoplastics, we developed additional lists of related terms and text patterns and identified all the posts that contained exact matches to these patterns (Multimedia Appendices 2-3) after lowercasing all the posts. Posts were deemed relevant and included for analyses if they were (1) in a thread whose title or initial question contained an exact pattern match (Multimedia Appendix 2) or (2) the post itself contained an exact pattern match to a very specific oculoplastics-related term (Multimedia Appendix 3). Posts that were not part of a relevant thread were subject to more stringent inclusion criteria because the original topic of the thread did not necessarily pertain to oculoplastics. This filtering algorithm ensures that the data set is relevant and tailored and is not influenced by the proprietary search algorithm of the platform.

Patterns required for inclusion of posts allowed for some variability in human language, for example, the two patterns “%upper lid%eye” and “eye%upper lid%” (“%” denotes 0 or more of any character) match a subset of posts expressing one’s upper eyelid, such as “my eye hurts, and my upper lid...” and “my upper lid droops, and my eye keeps twitching,” without deeming posts containing solely “upper lid” as relevant, such as “the upper lid of my jar....” After excluding irrelevant posts, we extracted the username, user type (doctor or patient), self-reported age, sex, registration date to the MedHelp community, and user location from each user profile. All data were stored in an SQLite relational database [29]. The scripts used to extract threads, posts, and users and the detailed instructions on how to use them can be found in our repository [30].

### Approach to Natural Language Understanding Processing

**Figure 2:**
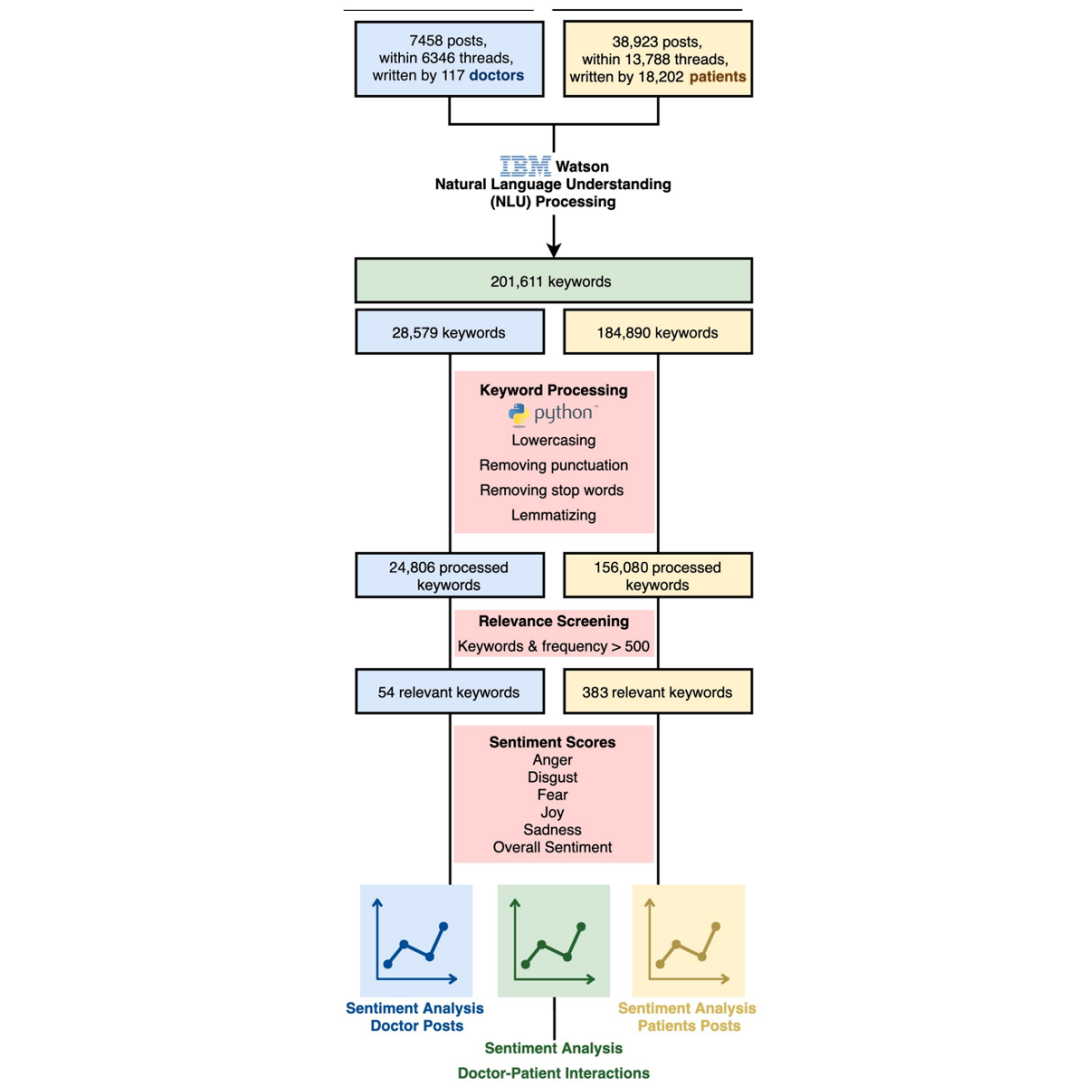
Flowchart describing keyword processing and sentiment analysis.

The approach to NLP and sentiment analysis is presented in Figure 2 [31]. We used IBM’s Watson Natural Language Understanding (NLU; IBM Cloud Natural Language Understanding V1, version 2019-07-12) [32] to perform sentiment and emotion analyses on the free text of every included forum post. The Watson machine learning system reads and understands the semantics of free text by breaking down sentences structurally, grammatically, and contextually through various linguistic models and algorithms. The results that were returned included a sentiment score for the full document (ie, the full text of a single post) and for each keyword extracted by the IBM Watson algorithm and emotion scores for anger, sadness, joy, fear, and disgust at both the post and keyword levels. These keywords include important words, entities, and phrases from each post. Sentiment scores ranged from −1 to +1 on an arbitrary linear scale of intensity and were negative (less than 0), neutral (0), or positive (greater than 0). For each emotion, a score was given in the form of a percentage of likelihood, ranging from 0 to 1, where 0 represents the certain absence of the emotion in question and 1 represents the definite presence of the emotion.

### NLU Keywords Processing

Related keywords generated by the IBM Watson NLU program were processed using a Jupyter Notebook [33] with Natural Language Toolkit (NLTK) [34], NumPy [35], and Pandas [36] libraries. The following transformations were applied to each keyword: lowercasing, punctuation removal, stop word deletion (eg, prepositions and conjunctions), and lemmatization [37] (morphological destructuring that allows words to be stripped down to their root word, eg, “oculoplastics” into “oculoplastic”).

### NLU Keywords Selection and Categorization

Among the keywords with a frequency higher than 500, manual verification was performed to merge the keywords with the same semantic meaning. These keywords were then classified into various categories (groups and subgroups). For example, the “people” group encompasses multiple subgroups including the “eye care provider” subgroup, which in turn contains the fully processed keywords “ophthalmologist” and “optometrist.” However, keywords with a questionable relevancy to the clinical field and keywords with a general meaning (eg, “thing,” “thought,” and “name”) were excluded from the analysis.

### Sentiment Scores Statistical Analysis

We used Python to aggregate and calculate the mean and standard deviation of each keyword’s associated sentiment and emotion scores (sentiment, sadness, fear, anger, joy, and disgust scores). Three examples of the analyses were performed with the results. We performed a summary of statistics by keyword grouping to determine significant trends among the chosen clinical categories. We also analyzed the data by forum subgroups (eg, posts in the *Eye Care* forum vs posts in the *Neurology* forum). We also compared the sentiment associated with the posts written by the patient before a doctor replied with the patient’s posts written after a doctor replied.

## Results

### Results From Data Extraction

#### Threads Extraction and Filtering

Searching the 300 forums (including ongoing communities, discontinued forums, and user-made groups) on MedHelp using 39 oculoplastics-related search terms resulted in 22,623 discussion threads (Multimedia Appendix 1). The screening for irrelevant threads resulted in the exclusion of 6 duplicate threads, 330 threads found in animal-related forums, and 92 threads containing the search term used exclusively as an idiom. Table 1 highlights threads containing the common idioms associated with the initial search term lists and excluded forums (*Animal Health—General*, *Animal Lovers Group*, *Animal-Surgery*, *Birds*, *Cats*, *Dogs*), as well as example text from the excluded threads and the associated number of threads deleted.

**Table 1:** Examples of excluded posts because of idiomatic language or reference to animals.

Idiom or forum name			Description		Threads deleted, n (%)			Example text from excluded threads
**Idiom**								
	(1) Raise an *eyebrow*^a^; (2) raise an *eye brow*; (3) raise *eyebrows*	This idiom is used to convey awe, consternation, or disbelief.		(1) 51 (100); (2) 2 (100); (3) 18 (64)			“I may be just freaking out but it does raise an *eyebrow.*”	
	(1) Bat an *eyelid*; (2) bat an *eye lid*	This idiom is used to show an emotional reaction.		(1) 20 (100); (2) 1 (100)			“And the doctor, like me, has seen so many she’s not going to bat an *eyelid*!”	
**Forum**								
	Animal Health—General	This forum is used to answer questions related to general pet health (treatment, parasites, infectious disease, etc).		56 (100)			“My 3 year old boxer has one *eye* that seems to droop and is a little redder than normal. [...] It has always been that way it could be a congenital abnormality such as *entropion*.”	
	Animal Lovers Group	This forum was previously used to chat about anything related to pets and animals.		1 (100)			“Birds are wonderful. In this state, they seem to *frown* on folks feeding them in the park too, it really irritates me, what would our world be like without those lovely creatures singing their happy song to us, I love them.”	
	Animal-Surgery	This forum was previously used to have questions answered by a veterinarian from PetDocsOnCall on all questions regarding animal surgery.		2 (100)			“My dog has ingrown *eyelashes*”	
	Birds	This forum was used to answer questions about pet birds.^b^		5 (100)			“My three year old peacock has cloudy *eyes.* One *eye* in particular, the lid seems to linger and appears to bulge (slightly) when looking at him straight.”	
	Cats	This forum was used to answer questions about pet cats.^b^		113 (100)			“I don’t know what my cat has got into but his left *eye* has been watering really bad and is red inside. It is now red on the right *eye* but just around where the *lashes* would be.”	
	Dogs	This forum was used to answer questions about pet dogs.^b^		153 (100)			“Lumps on dogs *eye lid*”	

^a^Words referring to ophthalmology are italicized.

^b^These forums used to have questions answered by a veterinarian.

#### Posts Extraction and Filtering

After filtering the threads, 129,393 posts associated with the resulting 22,195 threads remained, which then underwent additional layers of filtering for inclusion and exclusion (Figure 1). Posts from 13,239 of the 22,195 threads were considered relevant and were therefore included because the thread title or question contained a relevant oculoplastic term (Multimedia Appendix 2), which resulted in 44,882 included posts. An additional 1499 individual posts from 1090 other discussion threads also contained oculoplastic-related keywords (Multimedia Appendix 3) and were therefore included in the analysis. The final corpus was composed of 46,381 posts within 14,329 threads, which were written between January 1, 1995, and December 18, 2019, in 273 forums.

#### User Extraction

These 46,381 posts were written by 18,319 users from 1995 to 2019. More specifically, 7458 posts (within 6346 threads) were written by 117 doctors, and 38,923 posts (within 13,788 threads) were written by 18,202 patients. Overall, 20.19% (3699/18,319) of users were male patients, 38.33% (7022/18,319) were female patients, 40.84% (7481/18,319) of the patients did not specify their sex, 0.41% (75/18,319) were male doctors, and 0.23% (42/18,319) were female doctors. A total of 5642 patients were included in this study. Their ages varied from 10 to 96 years, with an average of 44.8 years. A total of 6704 patients indicated their location (city, state, and/or country).

### Results From Keyword Processing

#### Keyword Extraction

Keyword extraction, sentiment analysis, and emotion analysis were performed using the IBM Watson NLU service, which generated 201,611 unique raw keywords, including 28,579 keywords from posts written by doctors and 184,890 keywords from posts written by patients, with some keywords common to both sets of posts (Figure 2). Further processing using the NLTK Python library grouped related keywords, resulting in 24,806 keywords from doctors’ posts and 156,080 keywords from patients’ posts. For instance, “eyes” became “eye,” “eyelids” became “eyelid,” and “eye lashes” became “eye lash.”

#### Keyword Selection and Categorization

**Figure 3:**
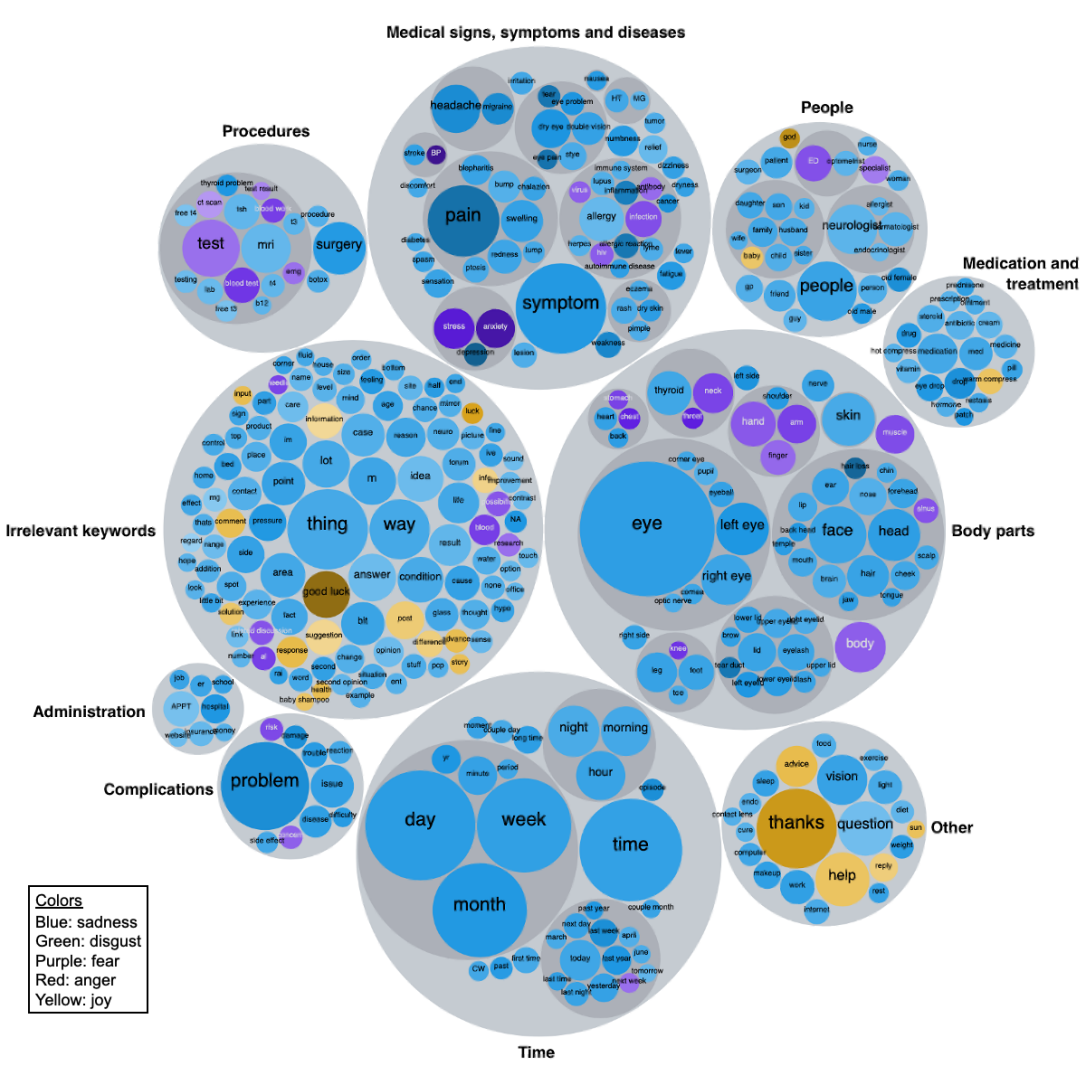
Nested bubble chart showing the top 500 keywords associated with patient posts and grouped into clinically relevant categories. The size of each bubble is proportional to the frequency of the keyword. The color of each bubble represents the most likely emotion associated with the keyword. The shade of each bubble is proportional to the likelihood of the emotion score; emotions that are more likely are in darker bubbles. APPT: appointment; BP: blood pressure; ED: eye doctor; HP: hypothyroidism; MG: myasthenia gravis.

Keywords that occurred at least 500 times in the corpus were included for analysis; 383 keywords were from patients’ posts and 54 keywords were from doctors’ posts. We grouped these keywords into nine relevant categories: body parts; medical signs, symptoms, and diseases; people; medication and treatment; procedures; complications; administration; aggravating and relieving factors; and others. Some of these categories were then subdivided into more precise clinical concepts. For example, the broad category *body parts* contained keywords related to the head, neck, upper limbs, thorax, and lower limbs. The category *medical signs, symptoms, and diseases* was subdivided by specialty (oculoplastics, ophthalmology, psychiatry, neurology, endocrinology, integumentary, immunology, cardiology, and gastroenterology). The *people* category contained references to eye care doctors, nonocular medical specialists, surgeons, family doctors, family members, friends, and other health care professionals (Figure 3) [38].

Similar keywords that were aggregated include the following examples: “itch” encompassing both “itch” and “itching,” “diagnosis” replacing “dx” and “diagnosis,” “eyelid” including both “eyelid” and “eye lid,” “eyebrow” (“eye brow” and “eyebrow”), “twitch” (“twitch” and “twitching”), “treatment” (“tx” and “treatment”), “non specified doctor” (“doctor,” “doc,” “dr,” “physician,” and “md”), and “ophthalmologist” (“ophthalmologist” and “ophthamologists” [sic]).

### Sentiment and Emotion Analysis

**Figure 4:**
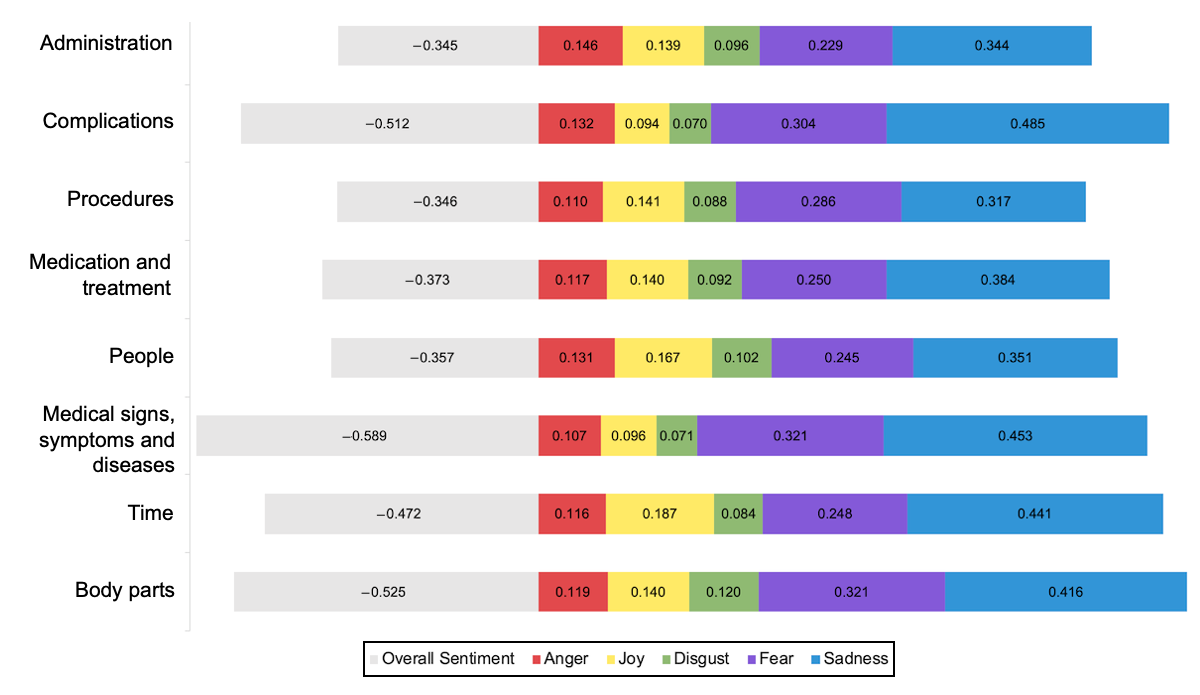
Top 8 groupings and their respective overall sentiment and emotion scores. The overall sentiment score reflects a positive, neutral, or negative sentiment, whereas the emotion score (anger, disgust, fear, joy, and sadness) represents how likely (%) the emotion is to be present.

Summary statistics were therefore performed using keyword groupings (Figures 3 and 4). Medical signs, symptoms, and diseases had the lowest overall sentiment scores (−0.598). Complications were highly associated with sadness (likelihood sadness score of 0.485). Forum posts mentioning body parts were related to sadness (likelihood sadness score of 0.416) and fear (likelihood fear score of 0.321). Administration was the category with the highest anger score (0.146).

We further analyzed sentiments and emotions by the forum subgroup. We compared the most popular forums among each other by analyzing the sentiment and emotion scores of their posts (Multimedia Appendix 4). All 6 forums had an overall negative sentiment score; the most negative one being the *Neurology* forum with a score of −0.438. The *Undiagnosed Symptoms* forum had the highest sadness score (0.448). The least likely fearful posts were those from the *Eye Care* forum, with a score of 0.260.

We also analyzed all the posts from users who asked questions (ie, initiated new threads) on MedHelp. These posts were divided into two categories: the pre–doctor reply group and the post–doctor reply group. The pre–doctor reply group included all the questions, the self-replies, and replies to other users written by the initial user before a doctor replied. The post–doctor reply group included all the other posts written by the initial user after the first doctor replied. As seen in Table 2, the overall sentiment score is much more negative before the doctor replied. We can also see shifts in the emotion scores: anger, disgust, fear, and sadness decreased in likelihood whereas joy was expressed slightly more likely after the doctor replied.

**Table 2:** Difference in sentiment and emotion scores between the posts written before and after a doctor replied.

Posts analyzed		Pre–doctor reply group	Post–doctor reply group	Difference (post − pre)
**Posts expressing the following sentiment**				
	Negative, n (%)	1553 (92.22)	1260 (49.55)	−42.67%
	Neutral, n (%)	11 (0.65)	110 (4.33)	+3.67%
	Positive, n (%)	120 (7.13)	1172 (46.09)	+38.97%
**Scores**				
	Overall sentiment	−0.557	0.0268	+0.584
	Anger	0.143	0.109	−0.0334
	Disgust	0.126	0.0740	−0.0505
	Fear	0.364	0.233	−0.130
	Joy	0.308	0.348	+0.0391
	Sadness	0.5210	0.335	−0.186

## Discussion

### Innovation

This is the first paper providing a detailed methodology for preparing unstructured text data from web-based health discussion forums related to ophthalmology for sentiment and emotion analyses. We detailed the steps performed to quantify patients’ and doctors’ sentiments from web-based discussion forums: searching results, extracting a data corpus of threads and posts, cleaning the data, analyzing text using IBM Watson NLU, and aggregating and processing the important keywords from each post. Our goal was to explain these key steps and highlight the applicability of our methods to the field of medicine and the factors to consider in the process, notably the selection of search terms; understanding the latter’s different linguistic usages (eg, idioms); the adequate consideration of different forums; and the establishment of robust criteria for data cleaning, aggregation, and grouping of posts and keywords (eg, lowercasing, punctuation removal, and lemmatization). Our approach highlights the importance of considering the unique structure of discussions within web-based health forums, distinguishing between physician and patient posts and analyzing idiomatic language usage to determine text relevance in infoveillance studies, which we found to be important steps not commonly detailed in previous studies of web-based health forums [39,40].

### Medical Application

Analyses examining groupings (eg, administration; complications; procedures; medication and treatment; people; medical signs, symptoms, and diseases; time; and body parts), forum subgroups (eg, eye care, neurology, dermatology, thyroid disorders, multiple sclerosis, and undiagnosed symptoms), and patient-doctor interactions can enable researchers to provide key recommendations to physicians. In the oculoplastics data set, patients had a highly negative overall sentiment score and emotion score (anger, disgust, fear, and sadness) before the doctor replied (Table 2). To improve patient satisfaction, health care professionals can address their concerns by adapting their responses to the patients’ sentiments and emotions. These sentiments and emotions can be further broken down by grouping and forums. Each grouping can be addressed with different solutions, such as reducing appointment and waiting time; explaining medical signs, symptoms, and diseases; and reassuring patients’ concerns regarding specific procedures and body parts (Figure 4). Each forum’s scores indicate how the corresponding health care team (eg, neurology, endocrinology, and ophthalmology) must communicate with patients to better manage different emotions, different emotions by predominantly addressing patients’ sadness, disgust, fear, or even joy (Multimedia Appendix 4).

### Challenges and Factors to Consider

Several issues must be carefully considered when gathering data from internet sources and unstructured free text to ensure relevance to the desired topic. First, the selection of the search terms is critical when analyzing web-based content. A deep understanding of the chosen field along with its related terms (eg, symptoms, complications, and subfields) is crucial to establish a complete list that encompasses all the possible relevant thread discussions. Second, a thorough understanding of the linguistic usages of the search terms is critical for establishing adequate data cleaning algorithms (eg, removal of threads containing the search terms exclusively used as idioms and consideration of human speech variance in the filtering algorithm). There are many eye-related idioms in the English language that must be considered when analyzing web-based text for ophthalmology-related insights (eg, “bat an eyelid”); every specialty will have its own unique set of idioms related to anatomical parts or functions (eg, “break my heart” and “take my breath away”) that must be taken into consideration. The results can also differ according to the terms’ specificity: broader terms (eg, eyelids, eyebrows, and oculoplastics) encompass the oculoplastics field, whereas more specific terms (eg, blepharitis, entropion, and ectropion) refer to specific medical conditions in this field. It is recommended to choose all relevant search terms (broad and specific) to ensure exhaustive results. However, a robust and tailored filtering algorithm must be established to ensure a relevant data set that is not influenced by the initial results returned by any proprietary search algorithm for any platform.

Indeed, every social media platform will have individual and proprietary search functions that may retrieve information irrelevant to the original query. Therefore, a careful and tailored process for further filtering is required to remove irrelevant results. Key decisions must be made on the filtering process (filtering by topic title, discussion thread, and/or individual post content). Establishing these filtering guidelines is crucial to ensure that the content of the posts selected is relevant and that the posts discarded do not contain relevant information. Basing the filtering algorithm on the relevancy of the thread topic allows for this methodology to be applied to many other social media platforms that often contain similar data structures (eg, on Facebook, Twitter, and Instagram, a main post (topic or title) is followed by comments (replies) related to the initial topic).

Furthermore, the scope of the search must also be evaluated. Depending on the topic selected, forums outside of those dedicated to the primary specialty may also need to be included. In our study, we considered a wide variety of MedHelp forums outside the eye care forums as oculoplastics is a field at the intersection of ophthalmology and plastic surgery. The *Eye Care* forum is only one of the 273 forums that contained our relevant threads and posts (ie, the *Cosmetic Surgery*, *Dermatology*, *Neurology*, and *Thyroid Disorders* forums). As we took all MedHelp forums into account during the extraction process, more constraints had to be established. For example, all forums related to animal care needed to be excluded.

After carefully selecting individual posts on which sentiment analysis is performed, the keywords extracted by the program will be numerous and lexically repetitive. Therefore, care must be taken to normalize the results originally sourced from free text. Using NLP tools to process and group the keywords with the same clinical meaning is a crucial step to ensure that the analysis is performed on uniform and clean data. To facilitate the grouping of related processed keywords, following a systematic method, such as ours (all keywords with a frequency greater than 500 and keyword categorization by 2 reviewers), prevents biases from being induced into the sentiment analysis and results.

### Limitations

Although the effects of users’ spatiotemporal characteristics on sentiment analyses in MedHelp have not been evaluated yet, studies have shown that these features can bias the results of sentiment analysis derived from tweets. Gore et al showed that sentiment analysis can yield biased measures related to population demographics at the municipal, state, and national levels [41]. Another study demonstrated that an individual’s location throughout the day can also affect their tweets’ sentiment [42]. These issues can be addressed by assessing the population represented by posts on the web. In the case of Twitter, only 15% of adults on the web regularly use Twitter, and those aged 18-29 years and minorities tend to be more highly represented on Twitter than in the general population [43]. Although it is unclear what effect these spatial, temporal, and demographic effects may have on sentiment and emotion reflected in forum posts, they have the potential to affect these findings. We acknowledge that not all patients will rely on web-based forums to discuss their medical concerns or receive expert advice, especially the most vulnerable (older adults, minority, and socioeconomic groups).

### Conclusions

Despite these limitations, the internet is a major source of health-related information that is underused [44]. In this paper, we describe an accessible, quick, and robust approach to sentiment analysis of patient data in social media that is relevant to a chosen medical topic, such as oculoplastics, and highlight the technical challenges encountered when preparing and analyzing the data. Regardless of the clinical questions examined, important factors to be considered during the application of this methodology include assessing the scope of the research; determining search terms and understanding their different linguistic usages; and implementing selection, filtering, and processing criteria for posts and keywords tailored to the results. This emerging methodology can be used as a valuable guide for clinicians and researchers who want to better understand patient attitudes toward and patient satisfaction with particular fields and procedures. The analysis of web-based forum discussions can be a quick, efficient, and robust method for gathering unstructured, diverse, and detailed opinions relevant to a chosen medical topic such as oculoplastics.

## Appendix

Multimedia Appendix 1 Initial search terms.

Multimedia Appendix 2 List of patterns used to filter threads based on their titles or initial questions.

Multimedia Appendix 3 List of patterns used to filter posts based on their content.

Multimedia Appendix 4 Top 6 forums and their respective overall sentiment and emotion scores. The overall sentiment score reflects a positive, neutral, or negative sentiment, whereas the emotion score (anger, disgust, fear, joy, and sadness) represents how likely (%) the emotion is to be present. These forums had the highest number of posts and threads (displayed in the table).

## Abbreviations

NLP: natural language processing
NLTK: Natural Language Toolkit
NLU: Natural Language Understanding
