# Supplementary material for: Determination of Patient Sentiment and Emotion in Ophthalmology: Infoveillance Tutorial on Web-Based Health Forum Discussions
Source: J Med Internet Res. 2021 May 17;23(5):e20803. doi: 10.2196/20803 (PMC8167608; doi:10.2196/20803)
Supplement: Multimedia Appendix 1 [file jmir_v23i5e20803_app1.docx]

**Supplementary Table 1.** Initial Search Terms

| Unique Identifier | Search Term Used |
| --- | --- |
| 1 | blepharitis |
| 2 | blepharoplasty |
| 3 | botox eye |
| 4 | botox eyes |
| 5 | canthoplasty |
| 6 | crow's feet |
| 7 | double eye lid |
| 8 | double eye lids |
| 9 | double eyelid |
| 10 | double eyelids |
| 11 | ectropion |
| 12 | entropion |
| 13 | eye brow |
| 14 | eye brows |
| 15 | eye lid |
| 16 | eye lids |
| 17 | eyebrow |
| 18 | eyebrows |
| 19 | eyelid |
| 20 | eyelid filler |
| 21 | eyelid lift |
| 22 | eyelid rejuvenation |
| 23 | eyelids |
| 24 | lower eyelid |
| 25 | lower lid |
| 26 | lower lid blepharoplasty |
| 27 | lower lid lift |
| 28 | oculoplastic |
| 29 | oculoplastics |
| 30 | ptosis |
| 31 | thyroid eye lid |
| 32 | thyroid eye lids |
| 33 | thyroid eyelid |
| 34 | thyroid eyelids |
| 35 | thyroid lid |
| 36 | upper eyelid |
| 37 | upper lid |
| 38 | upper lid blepharoplasty |
| 39 | upper lid lift |
