# Supplementary material for: Determination of Patient Sentiment and Emotion in Ophthalmology: Infoveillance Tutorial on Web-Based Health Forum Discussions
Source: J Med Internet Res. 2021 May 17;23(5):e20803. doi: 10.2196/20803 (PMC8167608; doi:10.2196/20803)
Supplement: Multimedia Appendix 2 [file jmir_v23i5e20803_app2.docx]

**Supplementary Table 2.** List of patterns used to filter the threads based on their title or initial question

| **Value** | | |
| --- | --- | --- |
| %bag%eye% | %enucleation% | %eye%upper lid% |
| %bleph% | %eye%evisceration% | %eyebrow% |
| %botox%eye% | %evisceration%eye% | %eyelash% |
| %bottom lid%eye% | %eye%extenteration% | %eyelid% |
| %brow lift% | %extenteration%eye% | %hughes procedure% |
| %brow plasty% | %eye brow% | %intracanalicular plug% |
| %browplasty% | %eye lash% | %lid%thyroid% |
| %canthal% | %eye lid% | %lower lid%eye% |
| %canthectomy% | %eye lift% | %meibo% |
| %cantholysis% | %eye%bag% | %oculoplast% |
| %canthopexy% | %eye%botox% | %plug%eye% |
| %canthoplasty% | %eye%bottom lid% | %ptosis% |
| %canthorraphy% | %eye%ectropion% | %puffiness%eye% |
| %canthotomy% | %eye%entropion% | %puffy%eye% |
| %canthus% | %eye%lidplasty% | %punctal plug% |
| %crow's feet% | %eye%lower lid% | %tarsorrhaphy% |
| %crows feet% | %eye%plug% | %tear duct% |
| %dacryo% | %eye%puffiness% | %thyroid%lid% |
| %ectropion%eye% | %eye%puffy% | %top lid%eye% |
| %entropion%eye% | %eye%top lid% | %upper lid%eye% |
