# Supplementary material for: Determination of Patient Sentiment and Emotion in Ophthalmology: Infoveillance Tutorial on Web-Based Health Forum Discussions
Source: J Med Internet Res. 2021 May 17;23(5):e20803. doi: 10.2196/20803 (PMC8167608; doi:10.2196/20803)
Supplement: Multimedia Appendix 3 [file jmir_v23i5e20803_app3.docx]

**Supplementary Table 3.** List of patterns used to filter posts based on their content

| **Value** | | |
| --- | --- | --- |
| %bleph% | %dacryo% | %eye%lid%thyroid% |
| %botox%eye%lid% | %ectropion%eye% | %eye%plug% |
| %brow lift% | %entropion%eye% | %eyelid plasty% |
| %brow plasty% | %enucleation% | %eyelidplasty% |
| %browplasty% | %evisceration%eye% | %hughes procedure% |
| %canthal% | %extenteration%eye% | %intracanalicular plug% |
| %canthectomy% | %eye lid plasty% | %meibo% |
| %cantholysis% | %eye lidplasty% | %oculoplast% |
| %canthopexy% | %eye lift% | %ptosis% |
| %canthoplasty% | %eye plug% | %punctal plug% |
| %canthorraphy% | %eye%ectropion% | %tarsorrhaphy% |
| %canthotomy% | %eye%entropion% | %tear duct% |
| %canthus% | %eye%evisceration% | %thyroid%eye%lid% |
| %crow's feet% | %eye%extenteration% |  |
| %crows feet% | %eye%lid%botox% |  |
