# Supplementary material for: Determination of Patient Sentiment and Emotion in Ophthalmology: Infoveillance Tutorial on Web-Based Health Forum Discussions
Source: J Med Internet Res. 2021 May 17;23(5):e20803. doi: 10.2196/20803 (PMC8167608; doi:10.2196/20803)
Supplement: Multimedia Appendix 4 [file jmir_v23i5e20803_app4.docx]

**Supplementary Table 4.** Top six forums and their respective overall sentiment and emotion scores. The overall sentiment score reflects a positive, neutral or negative sentiment, while the emotions (anger, disgust, fear, joy, sadness) can be quantified from 0 (weakest) to 1 (strongest). These forums have the highest number of posts and threads (displayed in the table).

| Forum | | Eye Care | Neurology | Dermatology | Thyroid Disorders | Multiple Sclerosis | Undiagnosed Symptoms |
| --- | --- | --- | --- | --- | --- | --- | --- |
| Scores | Overall Sentiment | -0.306 | -0.438 | -0.275 | -0.251 | -0.216 | -0.407 |
|  | Anger | 0.116 | 0.139 | 0.133 | 0.127 | 0.151 | 0.123 |
|  | Disgust | 0.0928 | 0.0873 | 0.118 | 0.0870 | 0.105 | 0.104 |
|  | Fear | 0.260 | 0.369 | 0.281 | 0.273 | 0.298 | 0.350 |
|  | Joy | 0.248 | 0.294 | 0.269 | 0.328 | 0.389 | 0.277 |
|  | Sadness | 0.386 | 0.473 | 0.390 | 0.427 | 0.444 | 0.448 |
| Counts | Threads | 4987 | 1865 | 1531 | 638 | 426 | 403 |
|  | Posts | 13220 | 4762 | 4015 | 3121 | 2753 | 1445 |
